# Insights from the SNP analysis of TYMP gene linking MNGIE

**DOI:** 10.6026/973206300200261

**Published:** 2024-03-31

**Authors:** Najat Sifeddine, Lamiae Elkhattabi, Chaimaa Ait El Cadi, Al Mehdi Krami, Khadija Mounaji, Bouchra el khalfi, Abdelhamid Barakat

**Affiliations:** 1 Laboratory of Genomics and Human Genetics, Institut Pasteur du Maroc, Casablanca, Morocco; 2 Laboratory of Physiology and Molecular Genetics, Department of Biology, Faculty of Sciences Ain Chock, Hassan II University of Casablanca, Casablanca, Morocco

**Keywords:** prediction, Mitochondrial Neurogastrointestinal Encephalopathy, Thymidine phosphorylase, nsSNPs, UTR, conservation, stability, molecular Modeling

## Abstract

TYMP gene, which codes for thymidine phosphorylase (TP) is also known as platelet-derived endothelial cell growth factor (PD-ECGF). TP plays crucial roles in nucleotide metabolism and angiogenesis. Mutations in the TYMP gene can lead to Mitochondrial Neurogastrointestinal Encephalopathy (MNGIE) syndrome, a rare genetic disorder. Our main objective was to evaluate the impact of detrimental non-synonymous single nucleotide polymorphisms (nsSNPs) on TP protein structure and predict harmful variants in untranslated regions (UTR). We employed a combination of predictive algorithms to identify nsSNPs with potential deleterious effects, followed by molecular modeling analysis to understand their effects on protein structure and function. Using 13 algorithms, we identified 119 potentially deleterious nsSNPs, with 82 located in highly conserved regions. Of these, 53 nsSNPs were functional and exposed, while 79 nsSNPs reduced TP protein stability. Further analysis of 18 nsSNPs through 3D protein structure analysis revealed alterations in amino acid interactions, indicating their potential impact on protein function. This will help in the development of faster and more efficient genetic tests for detecting TYMP gene mutations.

## Background:

The TYMP gene, responsible for producing thymidine phosphorylase (TP), is situated on chromosome 22q13.33 [1]. The TP, also known as platelet-derived endothelial cell growth factor, is an enzyme that plays a crucial role in catalyzing the reversible phosphorolysis of thymidine, deoxyuridine, and their analogs (excluding deoxycytidine). This enzymatic activity leads to the formation of the corresponding bases and 2-deoxy-D-ribose-1-phosphate (2-dR-1-P) [2]. In the human body, the expression of TP, also known as hTP, is noteworthy in several tissues, including macrophage-like cells, the placenta, lymph nodes, spleen, liver, lungs, and peripheral lymphocytes [3]. The TYMP is found to be overexpressed in various cancer types, encompassing head and neck [4], breast [5], lung [6], oral squamous carcinoma [7], esophageal [8], gastric [9], colorectal [10], bladder [11], prostate [12], ovarian [13], and cervical [14] cancers, among several others. Its biological effects in cancer are primarily characterized by strong pro-angiogenic [15] properties and anti-apoptotic activity [16]. Mutations within the TP gene are an uncommon source of mitochondrial neurogastrointestinal encephalomyopathy (MNGIE) [17]. Patients diagnosed with MNGIE display a marked reduction in TP activity, accompanied by a pronounced increase in the levels of thymidine and deoxyuridine in both the blood and tissues. This elevated presence of these substances has detrimental effects, causing disruption of mitochondrial DNA [1]. MNGIE presents a clinical profile characterized by a spectrum of symptoms, encompassing ptosis, ophthalmoparesis, gastrointestinal dysmotility, cachexia, peripheral neuropathy, myopathy, leukoencephalopathy, and lactic acidosis. Typically, the onset of MNGIE disease manifests before the age of 30 and sadly leads to the premature mortality of affected individuals between the ages of 20 and 40 [18]. This condition is intricately associated with the depletion and deletion of mitochondrial DNA (mtDNA), resulting from abnormalities in mitochondrial nucleoside/nucleotide metabolism [19]. Nonsynonymous SNPs (nsSNPs) located in coding regions can induce alterations in protein structure and/or function. Furthermore, in untranslated regions (UTRs), they frequently correlate with a range of diseases [20]. Identification of deleterious nsSNPs for most Human genes remains a major challenge in medical genetics. Therefore, it is of interest to identify deleterious SNPs that may affect the TP protein structure and/or function. In silico analyses conducted in this study not only advance our understanding of the impact of deleterious SNPs on TP protein structure and function but also lay a solid foundation for future experimental validations.

## Materials and Methods:

## Collection of nsSNPs:

Information regarding single nucleotide polymorphisms (SNPs) within the human TP gene was sourced from Ensembl (ensembl.org/), while the FASTA amino acid sequence of the TP protein (P19971) was retrieved from the UniProt database [21].

## Prediction of protein alterations:

The pathogenicity of each non-synonymous SNP (nsSNP) collected was predicted using PredictSNP [22], a resource consolidating predictions from various tools including SIFT (Sorting Intolerant from Tolerant) [23] , PolyPhen-2 (Polymorphism Phenotyping v2) [24], PhD-SNP [25], PANTHER [26], and SNAP [27]. SIFT employs sequence homology to predict the impact of coding mutations on protein function, while PolyPhen-2 assesses the influence of substitutions on protein structure and function based on physical properties. PhD-SNP utilizes support vector machine (SVM) methods to classify mutations as disease-causing or benign. PANTHER predicts pathogenicity based on evolutionary patterns. MAPP [24] predictions were based on physicochemical variation in sequence alignments.

## Sequence conservation:

ConSurf [28], a web-based algorithm, was employed to predict functionally important regions of the protein by estimating the degree of conservation of amino acid sites based on homology. The given score is between 1 and 9, representing the level of conservation of each amino acid. A score of 9 represents a highly conserved region, a score of 1 represents a highly variable region, and a score of 5 represents the average. This tool also reveals the type of residue in the giving position of the protein, which can be functional or structural and buried or exposed.

## Prediction of nsSNPs positions in different protein domains:

The InterPro tool [29] facilitates the prediction of domains and important sites of proteins based on functional analysis and classification into families. In this study, the InterPro tool was utilized to identify the positions of nsSNPs within different protein domains.

## System preparation and structural analysis:

The X-ray crystal structure of the human TP protein bound with thymine was retrieved from the Protein Data Bank (PDB) with a resolution of 2.31 Å (PDB ID 2j0f) [30]. Mutant protein structures were generated by substituting amino acids at corresponding positions, followed by energy minimization using the SPDB viewer tool [31] based on the GROMOS 96 force field.

## Prediction of the effect of nsSNPs located in the UTR region:

The 5' and 3' untranslated regions (UTRs) play crucial roles in post-transcriptional gene regulation, translation efficiency, mRNA subcellular localization, and stability. UTRScan [32] was employed to predict functional SNPs within these regions. This tool searches submitted sequences for motifs present in UTRsite, which derives data from UTRdb, a curated database updated through primary data mining and experimental validation.

## Results:

## SNP datasets:

A total of 513 non-synonymous SNPs (nsSNPs) were retrieved from the thymidine phosphorylase (TP) gene data available in Ensembl. Among these, 124 SNPs were identified in the 5' untranslated region (UTR), and 23 were located in the 3' UTR of the human TP gene.

## Prediction of deleterious nsSNPs:

**Figure 1:**
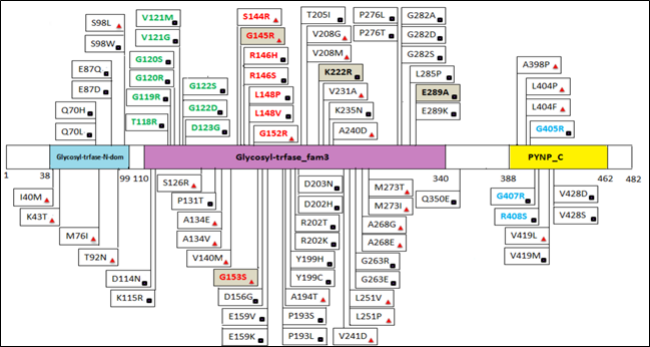
Location of different mutations in the human TYMP protein. Mutations that are structurally analyzed are subdivided into four groups: red =deleterious mutations in the glycine- rich loop; green=deleterious mutations in the active site loop; blue = deleterious mutations in the loop involved in stabilization of the dimmer interface; black square = exposed residues; buried residues = red triangle.

Out of 513 nsSNPs, 119 were predicted as deleterious by all integrated tools in PredictSNP and was selected for further analysis (Table 1).Conservation analysis using the Consurf web server revealed that out of 119 nsSNPs analyzed, 82 were located in highly conserved positions. Of these, 53 were identified as functional and exposed residues, while 29 were predicted to be buried. We selected only residues with a high degree of conservation (Figure 1).

## Prediction of different domains in TP:

The InterPro tool identified three domains within the TP protein: Glycosyl-Transferase-N-Domain (38-99), Glycosyl-Transferase-Fam3 (110-340), and PYNP-C (388-462). The distribution of highly conserved nsSNPs within these domains is illustrated in Figure 1.

## Impact of predicted deleterious mutations on tp stability:

Using I-Mutant 20, DUET, and MUpro web servers, it was found that 79 nsSNPs led to a decrease in the stability of TP. Table 2 summarizes these results.

## Structural analysis:

18 deleterious nsSNPs were selected for investigation. These chosen nsSNPs encompassed three variants located within residues crucial for thymine binding (R202K, R202T, and T118R), eight situated proximally to the active site (G120R, G120S, V121G, G122D, G122S, D123G, V208G, and V241D), two positioned within the loop involved in the closed conformation and stabilization of the dimer interface (G407R and R408S), and eight nsSNPs identified within the phosphate-binding site (S144R, G145R, R146H, R146S, and G153S). The substitution of arginine with threonine at position T118 resulted in the formation of a covalent bond with the thymine ligand and alterations in hydrophobic and hydrogen interactions compared to the native TP form. Mutations R202K and R202T led to the loss of hydrogen bonds with the thymine ligand and significant variations in hydrophobic interactions compared to the native form. The replacement of valine with guanine at conserved position 208 disrupted the hydrophobic interaction network compared to native TP. The V241D variant displayed destabilization in the hydrophobic domains, characterized by the acquisition of hydrophobic bonds with thymine and the loss of an interaction with IL241 compared to native TP. Additionally, the eight nsSNPs (S144R, G145R, R146H, R146S, L148P, L148V, G152R, and G153S) located in the phosphate-binding site, a highly conserved region, induced changes in both hydrophobic and hydrogen interactions.

## Prediction of deleterious nsSNPs in UTRs:

Using the UTRscan server, 32 SNPs were predicted to be functional in the internal ribosome entry site (IRES) within the 5' UTR of the TP gene. These functional SNPs are listed in Table 3.

## Discussion:

Mitochondrial neurogastrointestinal encephalomyopathy (MNGIE) is an uncommon autosomal recessive disorder that arises from mutations in the TP gene, causing dysfunction of the TP enzyme. TP functions as a homodimeric enzyme, with each subunit consisting of an α-helical domain (α domain) and a substantial α/β domain. These two domains are separated by a significant cleft that accommodates the active site for substrate binding [33]. Multiple reported missense mutations in the TP gene have been associated with the development of MNGIE [34]. A non-synonymous single nucleotide polymorphism (nsSNP) refers to a single-base alteration within the coding region of a gene, leading to the substitution of one amino acid for another in the corresponding protein. Investigating nsSNPs with functional relevance to diseases is a crucial goal in the fields of human molecular biology and medical research. Nevertheless, the sheer abundance of identified SNPs poses challenges in elucidating their biological significance through traditional wet laboratory experiments [35]. Over the past few decades, many studies have employed computational methods to assess the influence of mutations on protein structure and function. These approaches are effective in predicting whether a single-nucleotide polymorphism (SNP) has the potential to lead to a disease. In this work, different computational tools were used to identify the impact of nsSNPs on TP structure and stability. The total of 513 nsSNPs was analyzed by PredictSNP, and 119 of them were predicted to be the most deleterious. Additionally, we found 82 nsSNPs in highly conserved positions, including 54 nsSNPs in functional residues and 28 nsSNPs in structural residues.

**Figure 2:**
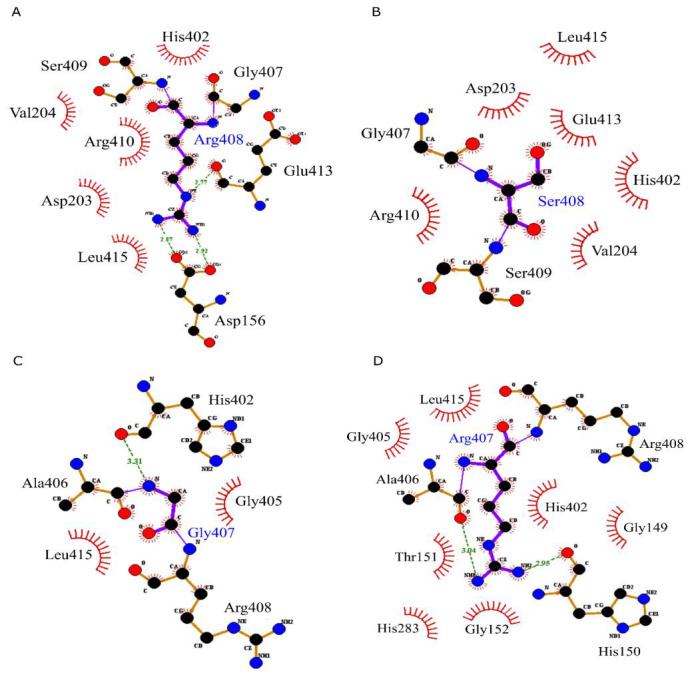
Residues contribute to the active site integrity and stability of the closed form. A:R408 (wild type-TYMP), B: S408 (variant type), C: G407 (wild type-TYMP), and D: R407 (variant type). Residues substituted are shown in blue.

The examination of the relationship between predicted amino acid alterations and the thermodynamic stability of proteins, as well as their impact on cellular stability and pathogenicity, implies that a decrease in stability may play a crucial role in the onset and progression of inherited diseases. It has been suggested that variants leading to the destabilization of proteins can disrupt their normal cellular functions, potentially giving rise to various genetic disorders [36]. In this study, the stability analysis revealed that the 79 nsSNPs reduced protein stability according to all the prediction tools employed. Only 18 deleterious nsSNPs were selected for molecular analysis based on their localization in the TP domains. This analysis focuses on the comparison of the differences in hydrogen bonds and hydrophobic interactions between the amino acids of the wild-type protein and its mutated forms. The deleterious SNPs (T118R, R202K, and R202T) contribute to thymine binding (Figure 2) and reveal a disruption of thymine binding (Table 3). The T118R mutation does not form a covalent bond with thymine. Instead, this mutation is associated with TP dysfunction, which can lead to the accumulation of thymine and other metabolites. This accumulation, caused by impaired TP enzymatic function, can contribute to the onset of MNGIE disease.

Deleterious nsSNPs localized in close proximity to the binding site of TP (G120R, G120S, V121G, G122D, G122S, D123G, V208M, V208G, and V241D) are in highly conserved positions. decrease TP's stability, leading to the disruption of the hydrogen and hydrophobic interaction, which may induce a change in the conformation of TP and may affect protein function. The two highly conserved nsSNPs (G407R and R408S) were localized in the important loop, which could potentially contribute to the integrity and stability of the closed conformation [37]. The structural property comparison between mutant forms and the WT protein showed a large change in the hydrophobic and hydrogen interactions (Figure 2). The residues Arg408, Ser409, and Arg410 make hydrogen bonds between the loop and the rest of the protein. Consequently, the two deleterious mutations are most likely to disrupt the structural and functional features of the WT protein. Eight nsSNPs (S144R, G145R, R146H, R146S, L148P, L148V, G152R, and G153S) are located in the glycine-rich loop, which has an important role in the binding of the catalytic phosphate [38]. Our in silico tests showed that the eight highlighted mutations are in a highly conserved region, decrease the TP's stability, and cause a vast variation in the residue-neighbor interaction compared to the native form (Table 2). We suggest that the studied mutations overall could affect TP catalytic efficiency through two possible mechanisms: decreasing the structural stability of the protein and reducing its binding affinity towards the essential cofactor PI. In addition, phosphate-binding domains of TP are responsible for the initiation of the closed conformation of the active site [39]. We suggested that substitution of glycine with either arginine or serine may cause MNGIE disease occurrence by disrupting phosphate binding or rendering the TP catalysis less effective. Multiple experimental studies involving the TYMP gene have demonstrated that some of the 19 non-synonymous single nucleotide polymorphisms (nsSNPs) are associated with significant manifestations in subjects of diverse ages and phenotypes. For instance, the R202T mutation was discovered in the TP gene of a 55-year-old Dutch woman who presented with ophthalmoplegia, severe bilateral ptosis, muscle atrophy (while maintaining normal muscle strength), intact sensory testing, and hypoactive or absent tendon reflexes. Additionally, she exhibited extensive leukoencephalopathy and polyphasic potentials in her leg muscles [40]. Similarly, the V208M mutation has been described in a 61-year-old Anglo-American woman who presented with a complex array of health issues, including pancreatitis, small intestine ileus, recurrent nausea with vomiting, early satiety, borborygmi, colonic diverticulosis, and hepatopathy. Furthermore, she experiences demyelinating sensorimotor polyneuropathy and has developed ptosis, progressive external ophthalmoplegia (PEO), optic atrophy, hearing loss, patchy leukoencephalopathy, short-term memory disturbances, occasional inappropriate behaviors, insulin-dependent diabetes mellitus, and renal cell carcinoma identified in patients who met the clinical criteria for mitochondrial neurogastrointestinal encephalomyopathy [39]. Moreover, the G145R mutation has been identified in patients presenting clinical symptoms consistent with mitochondrial neurogastrointestinal encephalomyopathy, originating from different regions, including Israel and Puerto Rico. Similarly, the G153S mutation has been identified in affected patients with clinical symptoms consistent with mitochondrial disorders [19]. All of the experimental data strongly align with the findings from our bioinformatic study, thus providing comprehensive evidence for and an explanation of the impact of deleterious nsSNPs on the TYMP gene. The IRES signal was described as a distinct RNA region that directly promotes the binding of 40S ribosomal subunits to mRNA without previous scanning [40]. The impairment of IRES sequences can deregulate mRNA translation and lead to various diseases or disease susceptibilities. Such as Charcot-Marie-Tooth disease (CMTX) [41], multiple myeloma [42], and Fragile X syndrome (FXS) [43]. We defined 32 SNPs in the IRES region; these SNPs may impair TP synthesis and lead to disease. The early detection of these potentially deleterious mutations in the TP gene could enable preventive intervention for individuals at risk, thereby paving the way for a reduction in the prevalence of MNGIE.

## Conclusion:

We identified 119 deleterious nsSNPs within the coding region of the TP gene. Out of them, 79 nsSNPs were predicted to perturb protein stability. Moreover, the structural analysis of 18 SNPs revealed disruption of the network of interaction compared to the native form of TP, which could destabilize the TP-Thymine complex and consequently induce the occurrence of the MNGIE disease. Additionally, we identified 32 functional SNPs in the 5' UTR, which could affect protein synthesis and may lead to diseases. This study lays the groundwork for future research aimed at experimentally validating the predictions of our in silico analysis, thereby paving the way for a better understanding of the underlying mechanisms of MNGIE.

## Data availability

All the datasets and structures generated for this study are available from the authors.

## Figures and Tables

**Table 1:** Software prediction and scores for the 119 deleterious nsSNP of the TYMP gene

	**PredictSNP prediction**	**MAPP prediction**	**PhD-SNP prediction**	**PolyPhen-1 prediction**	**PolyPhen-2 prediction**	**SIFT prediction**	**SNAP prediction**	**PANTHER prediction**
I40M	DELETERIOUS	DELETERIOUS	DELETERIOUS	DELETERIOUS	DELETERIOUS	DELETERIOUS	DELETERIOUS	DELETERIOUS
K43T	DELETERIOUS	DELETERIOUS	DELETERIOUS	DELETERIOUS	DELETERIOUS	DELETERIOUS	DELETERIOUS	DELETERIOUS
L49R	DELETERIOUS	DELETERIOUS	DELETERIOUS	DELETERIOUS	DELETERIOUS	DELETERIOUS	DELETERIOUS	DELETERIOUS
L49Q	DELETERIOUS	DELETERIOUS	DELETERIOUS	DELETERIOUS	DELETERIOUS	DELETERIOUS	DELETERIOUS	DELETERIOUS
Q70H	DELETERIOUS	DELETERIOUS	DELETERIOUS	DELETERIOUS	DELETERIOUS	DELETERIOUS	DELETERIOUS	DELETERIOUS
Q70L	DELETERIOUS	DELETERIOUS	DELETERIOUS	DELETERIOUS	DELETERIOUS	DELETERIOUS	DELETERIOUS	DELETERIOUS
M74K	DELETERIOUS	DELETERIOUS	DELETERIOUS	DELETERIOUS	DELETERIOUS	DELETERIOUS	DELETERIOUS	DELETERIOUS
L75R	DELETERIOUS	DELETERIOUS	DELETERIOUS	DELETERIOUS	DELETERIOUS	DELETERIOUS	DELETERIOUS	DELETERIOUS
M76I	DELETERIOUS	DELETERIOUS	DELETERIOUS	DELETERIOUS	DELETERIOUS	DELETERIOUS	DELETERIOUS	DELETERIOUS
G82R	DELETERIOUS	DELETERIOUS	DELETERIOUS	DELETERIOUS	DELETERIOUS	DELETERIOUS	DELETERIOUS	DELETERIOUS
E87D	DELETERIOUS	DELETERIOUS	DELETERIOUS	DELETERIOUS	DELETERIOUS	DELETERIOUS	DELETERIOUS	DELETERIOUS
E87Q	DELETERIOUS	DELETERIOUS	DELETERIOUS	DELETERIOUS	DELETERIOUS	DELETERIOUS	DELETERIOUS	DELETERIOUS
T92N	DELETERIOUS	DELETERIOUS	DELETERIOUS	DELETERIOUS	DELETERIOUS	DELETERIOUS	DELETERIOUS	DELETERIOUS
S98W	DELETERIOUS	DELETERIOUS	DELETERIOUS	DELETERIOUS	DELETERIOUS	DELETERIOUS	DELETERIOUS	DELETERIOUS
S98L	DELETERIOUS	DELETERIOUS	DELETERIOUS	DELETERIOUS	DELETERIOUS	DELETERIOUS	DELETERIOUS	DELETERIOUS
D114N	DELETERIOUS	DELETERIOUS	DELETERIOUS	DELETERIOUS	DELETERIOUS	DELETERIOUS	DELETERIOUS	DELETERIOUS
K115R	DELETERIOUS	DELETERIOUS	DELETERIOUS	DELETERIOUS	DELETERIOUS	DELETERIOUS	DELETERIOUS	DELETERIOUS
T118R	DELETERIOUS	DELETERIOUS	DELETERIOUS	DELETERIOUS	DELETERIOUS	DELETERIOUS	DELETERIOUS	DELETERIOUS
G119V	DELETERIOUS	DELETERIOUS	DELETERIOUS	DELETERIOUS	DELETERIOUS	DELETERIOUS	DELETERIOUS	DELETERIOUS
G119R	DELETERIOUS	DELETERIOUS	DELETERIOUS	DELETERIOUS	DELETERIOUS	DELETERIOUS	DELETERIOUS	DELETERIOUS
G120S	DELETERIOUS	DELETERIOUS	DELETERIOUS	DELETERIOUS	DELETERIOUS	DELETERIOUS	DELETERIOUS	DELETERIOUS
G120R	DELETERIOUS	DELETERIOUS	DELETERIOUS	DELETERIOUS	DELETERIOUS	DELETERIOUS	DELETERIOUS	DELETERIOUS
V121G	DELETERIOUS	DELETERIOUS	DELETERIOUS	DELETERIOUS	DELETERIOUS	DELETERIOUS	DELETERIOUS	DELETERIOUS
V121M	DELETERIOUS	DELETERIOUS	DELETERIOUS	DELETERIOUS	DELETERIOUS	DELETERIOUS	DELETERIOUS	DELETERIOUS
G122S	DELETERIOUS	DELETERIOUS	DELETERIOUS	DELETERIOUS	DELETERIOUS	DELETERIOUS	DELETERIOUS	DELETERIOUS
G122D	DELETERIOUS	DELETERIOUS	DELETERIOUS	DELETERIOUS	DELETERIOUS	DELETERIOUS	DELETERIOUS	DELETERIOUS
D123G	DELETERIOUS	DELETERIOUS	DELETERIOUS	DELETERIOUS	DELETERIOUS	DELETERIOUS	DELETERIOUS	DELETERIOUS
S126R	DELETERIOUS	DELETERIOUS	DELETERIOUS	DELETERIOUS	DELETERIOUS	DELETERIOUS	DELETERIOUS	DELETERIOUS
A130P	DELETERIOUS	DELETERIOUS	DELETERIOUS	DELETERIOUS	DELETERIOUS	DELETERIOUS	DELETERIOUS	DELETERIOUS
P131T	DELETERIOUS	DELETERIOUS	DELETERIOUS	DELETERIOUS	DELETERIOUS	DELETERIOUS	DELETERIOUS	DELETERIOUS
L133P	DELETERIOUS	DELETERIOUS	DELETERIOUS	DELETERIOUS	DELETERIOUS	DELETERIOUS	DELETERIOUS	DELETERIOUS
A134V	DELETERIOUS	DELETERIOUS	DELETERIOUS	DELETERIOUS	DELETERIOUS	DELETERIOUS	DELETERIOUS	DELETERIOUS
A134E	DELETERIOUS	DELETERIOUS	DELETERIOUS	DELETERIOUS	DELETERIOUS	DELETERIOUS	DELETERIOUS	DELETERIOUS
G137S	DELETERIOUS	DELETERIOUS	DELETERIOUS	DELETERIOUS	DELETERIOUS	DELETERIOUS	DELETERIOUS	DELETERIOUS
V140M	DELETERIOUS	DELETERIOUS	DELETERIOUS	DELETERIOUS	DELETERIOUS	DELETERIOUS	DELETERIOUS	DELETERIOUS
M142V	DELETERIOUS	DELETERIOUS	DELETERIOUS	DELETERIOUS	DELETERIOUS	DELETERIOUS	DELETERIOUS	DELETERIOUS
M142T	DELETERIOUS	DELETERIOUS	DELETERIOUS	DELETERIOUS	DELETERIOUS	DELETERIOUS	DELETERIOUS	DELETERIOUS
S144R	DELETERIOUS	DELETERIOUS	DELETERIOUS	DELETERIOUS	DELETERIOUS	DELETERIOUS	DELETERIOUS	DELETERIOUS
G145R	DELETERIOUS	DELETERIOUS	DELETERIOUS	DELETERIOUS	DELETERIOUS	DELETERIOUS	DELETERIOUS	DELETERIOUS
R146S	DELETERIOUS	DELETERIOUS	DELETERIOUS	DELETERIOUS	DELETERIOUS	DELETERIOUS	DELETERIOUS	DELETERIOUS
R146H	DELETERIOUS	DELETERIOUS	DELETERIOUS	DELETERIOUS	DELETERIOUS	DELETERIOUS	DELETERIOUS	DELETERIOUS
L148V	DELETERIOUS	DELETERIOUS	DELETERIOUS	DELETERIOUS	DELETERIOUS	DELETERIOUS	DELETERIOUS	DELETERIOUS
L148P	DELETERIOUS	DELETERIOUS	DELETERIOUS	DELETERIOUS	DELETERIOUS	DELETERIOUS	DELETERIOUS	DELETERIOUS
G152R	DELETERIOUS	DELETERIOUS	DELETERIOUS	DELETERIOUS	DELETERIOUS	DELETERIOUS	DELETERIOUS	DELETERIOUS
G153S	DELETERIOUS	DELETERIOUS	DELETERIOUS	DELETERIOUS	DELETERIOUS	DELETERIOUS	DELETERIOUS	DELETERIOUS
D156G	DELETERIOUS	DELETERIOUS	DELETERIOUS	DELETERIOUS	DELETERIOUS	DELETERIOUS	DELETERIOUS	DELETERIOUS
E159V	DELETERIOUS	DELETERIOUS	DELETERIOUS	DELETERIOUS	DELETERIOUS	DELETERIOUS	DELETERIOUS	DELETERIOUS
E159K	DELETERIOUS	DELETERIOUS	DELETERIOUS	DELETERIOUS	DELETERIOUS	DELETERIOUS	DELETERIOUS	DELETERIOUS
S160P	DELETERIOUS	DELETERIOUS	DELETERIOUS	DELETERIOUS	DELETERIOUS	DELETERIOUS	DELETERIOUS	DELETERIOUS
L177P	DELETERIOUS	DELETERIOUS	DELETERIOUS	DELETERIOUS	DELETERIOUS	DELETERIOUS	DELETERIOUS	DELETERIOUS
G181D	DELETERIOUS	DELETERIOUS	DELETERIOUS	DELETERIOUS	DELETERIOUS	DELETERIOUS	DELETERIOUS	DELETERIOUS
G186D	DELETERIOUS	DELETERIOUS	DELETERIOUS	DELETERIOUS	DELETERIOUS	DELETERIOUS	DELETERIOUS	DELETERIOUS
Q187K	DELETERIOUS	DELETERIOUS	DELETERIOUS	DELETERIOUS	DELETERIOUS	DELETERIOUS	DELETERIOUS	DELETERIOUS
S188R	DELETERIOUS	DELETERIOUS	DELETERIOUS	DELETERIOUS	DELETERIOUS	DELETERIOUS	DELETERIOUS	DELETERIOUS
S188C	DELETERIOUS	DELETERIOUS	DELETERIOUS	DELETERIOUS	DELETERIOUS	DELETERIOUS	DELETERIOUS	DELETERIOUS
L191P	DELETERIOUS	DELETERIOUS	DELETERIOUS	DELETERIOUS	DELETERIOUS	DELETERIOUS	DELETERIOUS	DELETERIOUS
P193S	DELETERIOUS	DELETERIOUS	DELETERIOUS	DELETERIOUS	DELETERIOUS	DELETERIOUS	DELETERIOUS	DELETERIOUS
P193L	DELETERIOUS	DELETERIOUS	DELETERIOUS	DELETERIOUS	DELETERIOUS	DELETERIOUS	DELETERIOUS	DELETERIOUS
A194T	DELETERIOUS	DELETERIOUS	DELETERIOUS	DELETERIOUS	DELETERIOUS	DELETERIOUS	DELETERIOUS	DELETERIOUS
A194V	DELETERIOUS	DELETERIOUS	DELETERIOUS	DELETERIOUS	DELETERIOUS	DELETERIOUS	DELETERIOUS	DELETERIOUS
Y199H	DELETERIOUS	DELETERIOUS	DELETERIOUS	DELETERIOUS	DELETERIOUS	DELETERIOUS	DELETERIOUS	DELETERIOUS
Y199C	DELETERIOUS	DELETERIOUS	DELETERIOUS	DELETERIOUS	DELETERIOUS	DELETERIOUS	DELETERIOUS	DELETERIOUS
R202K	DELETERIOUS	DELETERIOUS	DELETERIOUS	DELETERIOUS	DELETERIOUS	DELETERIOUS	DELETERIOUS	DELETERIOUS
R202T	DELETERIOUS	DELETERIOUS	DELETERIOUS	DELETERIOUS	DELETERIOUS	DELETERIOUS	DELETERIOUS	DELETERIOUS
D203H	DELETERIOUS	DELETERIOUS	DELETERIOUS	DELETERIOUS	DELETERIOUS	DELETERIOUS	DELETERIOUS	DELETERIOUS
D203N	DELETERIOUS	DELETERIOUS	DELETERIOUS	DELETERIOUS	DELETERIOUS	DELETERIOUS	DELETERIOUS	DELETERIOUS
T205I	DELETERIOUS	DELETERIOUS	DELETERIOUS	DELETERIOUS	DELETERIOUS	DELETERIOUS	DELETERIOUS	DELETERIOUS
V208G	DELETERIOUS	DELETERIOUS	DELETERIOUS	DELETERIOUS	DELETERIOUS	DELETERIOUS	DELETERIOUS	DELETERIOUS
V208M	DELETERIOUS	DELETERIOUS	DELETERIOUS	DELETERIOUS	DELETERIOUS	DELETERIOUS	DELETERIOUS	DELETERIOUS
S210R	DELETERIOUS	DELETERIOUS	DELETERIOUS	DELETERIOUS	DELETERIOUS	DELETERIOUS	DELETERIOUS	DELETERIOUS
K222R	DELETERIOUS	DELETERIOUS	DELETERIOUS	DELETERIOUS	DELETERIOUS	DELETERIOUS	DELETERIOUS	DELETERIOUS
G226A	DELETERIOUS	DELETERIOUS	DELETERIOUS	DELETERIOUS	DELETERIOUS	DELETERIOUS	DELETERIOUS	DELETERIOUS
K235N	DELETERIOUS	DELETERIOUS	DELETERIOUS	DELETERIOUS	DELETERIOUS	DELETERIOUS	DELETERIOUS	DELETERIOUS
A240D	DELETERIOUS	DELETERIOUS	DELETERIOUS	DELETERIOUS	DELETERIOUS	DELETERIOUS	DELETERIOUS	DELETERIOUS
V241D	DELETERIOUS	DELETERIOUS	DELETERIOUS	DELETERIOUS	DELETERIOUS	DELETERIOUS	DELETERIOUS	DELETERIOUS
L251V	DELETERIOUS	DELETERIOUS	DELETERIOUS	DELETERIOUS	DELETERIOUS	DELETERIOUS	DELETERIOUS	DELETERIOUS
L251P	DELETERIOUS	DELETERIOUS	DELETERIOUS	DELETERIOUS	DELETERIOUS	DELETERIOUS	DELETERIOUS	DELETERIOUS
L255P	DELETERIOUS	DELETERIOUS	DELETERIOUS	DELETERIOUS	DELETERIOUS	DELETERIOUS	DELETERIOUS	DELETERIOUS
V256G	DELETERIOUS	DELETERIOUS	DELETERIOUS	DELETERIOUS	DELETERIOUS	DELETERIOUS	DELETERIOUS	DELETERIOUS
V256D	DELETERIOUS	DELETERIOUS	DELETERIOUS	DELETERIOUS	DELETERIOUS	DELETERIOUS	DELETERIOUS	DELETERIOUS
G263E	DELETERIOUS	DELETERIOUS	DELETERIOUS	DELETERIOUS	DELETERIOUS	DELETERIOUS	DELETERIOUS	DELETERIOUS
G263R	DELETERIOUS	DELETERIOUS	DELETERIOUS	DELETERIOUS	DELETERIOUS	DELETERIOUS	DELETERIOUS	DELETERIOUS
A268E	DELETERIOUS	DELETERIOUS	DELETERIOUS	DELETERIOUS	DELETERIOUS	DELETERIOUS	DELETERIOUS	DELETERIOUS
A268G	DELETERIOUS	DELETERIOUS	DELETERIOUS	DELETERIOUS	DELETERIOUS	DELETERIOUS	DELETERIOUS	DELETERIOUS
L270P	DELETERIOUS	DELETERIOUS	DELETERIOUS	DELETERIOUS	DELETERIOUS	DELETERIOUS	DELETERIOUS	DELETERIOUS
M273I	DELETERIOUS	DELETERIOUS	DELETERIOUS	DELETERIOUS	DELETERIOUS	DELETERIOUS	DELETERIOUS	DELETERIOUS
M273T	DELETERIOUS	DELETERIOUS	DELETERIOUS	DELETERIOUS	DELETERIOUS	DELETERIOUS	DELETERIOUS	DELETERIOUS
P276L	DELETERIOUS	DELETERIOUS	DELETERIOUS	DELETERIOUS	DELETERIOUS	DELETERIOUS	DELETERIOUS	DELETERIOUS
P276T	DELETERIOUS	DELETERIOUS	DELETERIOUS	DELETERIOUS	DELETERIOUS	DELETERIOUS	DELETERIOUS	DELETERIOUS
G282S	DELETERIOUS	DELETERIOUS	DELETERIOUS	DELETERIOUS	DELETERIOUS	DELETERIOUS	DELETERIOUS	DELETERIOUS
G282A	DELETERIOUS	DELETERIOUS	DELETERIOUS	DELETERIOUS	DELETERIOUS	DELETERIOUS	DELETERIOUS	DELETERIOUS
G282D	DELETERIOUS	DELETERIOUS	DELETERIOUS	DELETERIOUS	DELETERIOUS	DELETERIOUS	DELETERIOUS	DELETERIOUS
L285P	DELETERIOUS	DELETERIOUS	DELETERIOUS	DELETERIOUS	DELETERIOUS	DELETERIOUS	DELETERIOUS	DELETERIOUS
E286G	DELETERIOUS	DELETERIOUS	DELETERIOUS	DELETERIOUS	DELETERIOUS	DELETERIOUS	DELETERIOUS	DELETERIOUS
E286K	DELETERIOUS	DELETERIOUS	DELETERIOUS	DELETERIOUS	DELETERIOUS	DELETERIOUS	DELETERIOUS	DELETERIOUS
E289A	DELETERIOUS	DELETERIOUS	DELETERIOUS	DELETERIOUS	DELETERIOUS	DELETERIOUS	DELETERIOUS	DELETERIOUS
E289K	DELETERIOUS	DELETERIOUS	DELETERIOUS	DELETERIOUS	DELETERIOUS	DELETERIOUS	DELETERIOUS	DELETERIOUS
C293R	DELETERIOUS	DELETERIOUS	DELETERIOUS	DELETERIOUS	DELETERIOUS	DELETERIOUS	DELETERIOUS	DELETERIOUS
G296R	DELETERIOUS	DELETERIOUS	DELETERIOUS	DELETERIOUS	DELETERIOUS	DELETERIOUS	DELETERIOUS	DELETERIOUS
L302S	DELETERIOUS	DELETERIOUS	DELETERIOUS	DELETERIOUS	DELETERIOUS	DELETERIOUS	DELETERIOUS	DELETERIOUS
G310R	DELETERIOUS	DELETERIOUS	DELETERIOUS	DELETERIOUS	DELETERIOUS	DELETERIOUS	DELETERIOUS	DELETERIOUS
L313P	DELETERIOUS	DELETERIOUS	DELETERIOUS	DELETERIOUS	DELETERIOUS	DELETERIOUS	DELETERIOUS	DELETERIOUS
A333E	DELETERIOUS	DELETERIOUS	DELETERIOUS	DELETERIOUS	DELETERIOUS	DELETERIOUS	DELETERIOUS	DELETERIOUS
L334R	DELETERIOUS	DELETERIOUS	DELETERIOUS	DELETERIOUS	DELETERIOUS	DELETERIOUS	DELETERIOUS	DELETERIOUS
F343C	DELETERIOUS	DELETERIOUS	DELETERIOUS	DELETERIOUS	DELETERIOUS	DELETERIOUS	DELETERIOUS	DELETERIOUS
Q350E	DELETERIOUS	DELETERIOUS	DELETERIOUS	DELETERIOUS	DELETERIOUS	DELETERIOUS	DELETERIOUS	DELETERIOUS
G387D	DELETERIOUS	DELETERIOUS	DELETERIOUS	DELETERIOUS	DELETERIOUS	DELETERIOUS	DELETERIOUS	DELETERIOUS
A398P	DELETERIOUS	DELETERIOUS	DELETERIOUS	DELETERIOUS	DELETERIOUS	DELETERIOUS	DELETERIOUS	DELETERIOUS
L404F	DELETERIOUS	DELETERIOUS	DELETERIOUS	DELETERIOUS	DELETERIOUS	DELETERIOUS	DELETERIOUS	DELETERIOUS
L404P	DELETERIOUS	DELETERIOUS	DELETERIOUS	DELETERIOUS	DELETERIOUS	DELETERIOUS	DELETERIOUS	DELETERIOUS
G405R	DELETERIOUS	DELETERIOUS	DELETERIOUS	DELETERIOUS	DELETERIOUS	DELETERIOUS	DELETERIOUS	DELETERIOUS
G407R	DELETERIOUS	DELETERIOUS	DELETERIOUS	DELETERIOUS	DELETERIOUS	DELETERIOUS	DELETERIOUS	DELETERIOUS
V419L	DELETERIOUS	DELETERIOUS	DELETERIOUS	DELETERIOUS	DELETERIOUS	DELETERIOUS	DELETERIOUS	DELETERIOUS
V419M	DELETERIOUS	DELETERIOUS	DELETERIOUS	DELETERIOUS	DELETERIOUS	DELETERIOUS	DELETERIOUS	DELETERIOUS
R408S	DELETERIOUS	DELETERIOUS	DELETERIOUS	DELETERIOUS	DELETERIOUS	DELETERIOUS	DELETERIOUS	DELETERIOUS
G428S	DELETERIOUS	DELETERIOUS	DELETERIOUS	DELETERIOUS	DELETERIOUS	DELETERIOUS	DELETERIOUS	DELETERIOUS
G428D	DELETERIOUS	DELETERIOUS	DELETERIOUS	DELETERIOUS	DELETERIOUS	DELETERIOUS	DELETERIOUS	DELETERIOUS
G434W	DELETERIOUS	DELETERIOUS	DELETERIOUS	DELETERIOUS	DELETERIOUS	DELETERIOUS	DELETERIOUS	DELETERIOUS
L459P	DELETERIOUS	DELETERIOUS	DELETERIOUS	DELETERIOUS	DELETERIOUS	DELETERIOUS	DELETERIOUS	DELETERIOUS

**Table 2:** Prediction of change in protein stability using I-Mutant2.0, MUpro and DUET

**SNP ID**	**substitution**	**Mupro**	**DDG**	**I-mutant**	**DUET**	**DDG**
rs935752285	I40M	decrease	-0.94	decrease	decrease	-0.995
rs752137335	K43T	decrease	-1.52	decrease	decrease	-1.645
rs772046185	Q70H	decrease	-0.98	decrease	decrease	-0.777
rs1190033207	Q70L	increase	0.076	increase	increase	0.816
rs1064792859	M76I	increase	0.166	decrease	decrease	-0.233
rs749827433	E87D	increase	0.16	decrease	decrease	-1.726
rs1361630544	E87Q	decrease	-0.58	decrease	decrease	-1.11
rs891107196	T92N	decrease	-0.62	increase	decrease	-1,37
rs758303113	S98L	increase	0,38	increase	decrease	-0.13
rs758303113	S98W	decrease	-0.3	increase	decrease	-1.35
rs1064792861	D114N	decrease	-1.25	decrease	decrease	-1.403
rs775841111	K115R	decrease	-0.87	decrease	decrease	-1.326
rs767829510	T118R	decrease	-0.943	decrease	decrease	-0.779
rs786205559	G119R	decrease	-1.37	decrease	decrease	-0.482
rs866018044	G119V	decrease	-1.36	decrease	decrease	-0.498
rs863224250	G120R	decrease	-0.92	decrease	decrease	-0.539
rs863224250	G120S	decrease	-0.92	decrease	decrease	-0.894
rs948237404	V121G	decrease	-1.65	decrease	decrease	-2.107
rs1159212438	V121M	decrease	-0.46	decrease	decrease	-0.998
rs1388735279	G122D	decrease	-0.16	decrease	decrease	-2.386
rs997046759	G122S	decrease	-0.61	decrease	decrease	-1.876
rs1452194925	D123G	decrease	-1.44	decrease	decrease	-0.682
rs749738967	S126R	decrease	-0.22	increase	decrease	-0.53
rs863224255	P131T	decrease	-0.85	decrease	decrease	-2.074
rs199901350	A134E	decrease	-0.352	decrease	decrease	-3.3385
rs199901350	A134V	decrease	-0.12	increase	decrease	-0.512
rs1207543220	V140M	decrease	-0.41	decrease	decrease	-1.688
rs751803871	S144R	decrease	-0.74	decrease	decrease	-0.763
rs121913037	G145R	decrease	-0.64	decrease	decrease	-0.897
rs188802138	R146H	decrease	-1.25	decrease	decrease	-1.313
rs1357734207	R146S	decrease	-1.14	increase	decrease	-1.239
rs1223312855	L148P	decrease	-1.75	decrease	decrease	-1.548
rs1022322270	L148V	decrease	-1.05	decrease	decrease	-0.543
rs765604179	G152R	decrease	0	decrease	decrease	-0.058
rs121913038	G153S	decrease	-1.29	decrease	decrease	-1.31
rs1064792863	D156G	decrease	-1.13	decrease	decrease	-0.777
rs777005301	E159K	decrease	-0.81	decrease	decrease	-0.311
rs863224251	E159V	decrease	-0.02	increase	decrease	-0.21
rs865961520	P193L	decrease	-0.06	decrease	decrease	-0.001
rs1236277429	P193S	decrease	-0.62	decrease	decrease	-1.627
rs923009362	A194T	decrease	-1.568	decrease	decrease	-1.727
rs1361750261	Y199C	increase	0.019	increase	decrease	-1.29
rs1434418446	Y199H	decrease	-0.4	decrease	decrease	-1.089
rs121913041	R202K	decrease	-1.57	decrease	decrease	-1.78
rs121913041	R202T	decrease	-1.54	decrease	decrease	-2.36
rs765932857	D203H	decrease	-1.01	decrease	decrease	-0.656
rs765932857	D203N	decrease	-0.71	decrease	decrease	-0.49
rs929350708	T205I	decrease	-0.5	decrease	decrease	0.96
rs1064792867	V208G	decrease	-4.1	decrease	decrease	-2.369
rs121913039	V208M	decrease	-0.7	decrease	decrease	-0.876
rs149977726	K222R	decrease	-0.76	decrease	decrease	-2.03
rs780762083	V231A	decrease	-3.5	decrease	decrease	-2.483
rs1372616333	K235N	decrease	-1.02	increase	decrease	-1.17
rs568773673	A240D	decrease	-0.7	decrease	decrease	-0.703
rs758373793	V241D	decrease	-0.86	decrease	decrease	-0.681
rs1487581282	L251P	decrease	-1.91	decrease	decrease	-2.028
rs1487581282	L251V	decrease	-1.07	decrease	decrease	-1.812
rs1199784789	G263E	decrease	-0.42	increase	decrease	-0.302
rs9628204	G263R	decrease	-0.46	decrease	decrease	-0.514
rs369432373	A268E	decrease	-0.61	decrease	decrease	-2.41
rs369432373	A268G	decrease	-1.39	decrease	decrease	-1.638
rs1190901146	M273I	decrease	-1.18	decrease	decrease	-0.665
rs1374755652	M273T	decrease	-2.22	decrease	decrease	-1.243
rs1253066888	P276L	increase	0.18	decrease	decrease	-0.216
rs1367937948	P276T	decrease	-0.78	decrease	decrease	-1.519
rs745699088	G282A	decrease	-0.3	decrease	decrease	-0.653
rs745699088	G282D	decrease	-0.14	decrease	decrease	-2.067
rs1297424973	G282S	decrease	-0.42	decrease	decrease	-1.442
rs121913042	L285P	decrease	-1.92	decrease	decrease	-1.848
rs946234163	E289A	Increase	0.21	decrease	decrease	-0,48
rs946234163	E289K	decrease	-0.86	decrease	decrease	-1.272
rs765023287	Q350E	decrease	-1.5	decrease	decrease	-1.706
rs1189525116	A398P	decrease	-1.73	increase	decrease	-0.827
rs995494519	L404F	decrease	-1.4	decrease	decrease	-1.495
rs1455473908	L404P	decrease	-2.27	decrease	decrease	-1.966
rs1272848697	G405R	decrease	-0.19	decrease	decrease	-0.354
rs863224254	G407R	decrease	-0.6	decrease	decrease	-0.622
rs898036117	R408S	decrease	-0.59	decrease	decrease	-2.137
rs1035967828	V419L	decrease	-0.18	decrease	decrease	-0.733
rs1035967828	V419M	decrease	-0.52	decrease	decrease	-1.174
rs1275136706	G428D	decrease	-0.25	decrease	decrease	-2.857
rs1064792874	G428S	decrease	-0.76	decrease	decrease	-2.158

**Table 3:** SNPs (UTR mRNA) that were predicted to be functionally significant by UTRscan

**SNP ID**	**Nucleotide change**	**UTR position**	**Functional element**
rs560027665	A/G	5'UTR	IRES signal
rs1462773364	G/T	5'UTR	IRES signal
rs999716676	G/A	5'UTR	IRES signal
rs1433459572	C/T	5'UTR	IRES signal
rs1297495211	G/C	5'UTR	IRES signal
rs1366475489	C/G	5'UTR	IRES signal
rs1235847706	G/A	5'UTR	IRES signal
rs1254678473	C/T	5'UTR	IRES signal
Ers1344522161	C/A	5'UTR	IRES signal
rs902800658	G/A	5'UTR	IRES signal
rs1025395522	A/G	5'UTR	IRES signal
rs895613086	A/C,G,T	5'UTR	IRES signal
rs1056976904	C/T	5'UTR	IRES signal
rs1433747756	G/C	5'UTR	IRES signal
rs1375747015	G/C,T	5'UTR	IRES signal
rs939884415	C/A	5'UTR	IRES signal
rs895147632	T/C	5'UTR	IRES signal
rs1055185491	C/T	5'UTR	IRES signal
rs1048500050	G/A	5'UTR	IRES signal
rs1409562773	C/T	5'UTR	IRES signal
rs541255050	C/G	5'UTR	IRES signal
rs1299593050	C/T	5'UTR	IRES signal
rs921271717	T/C	5'UTR	IRES signal
rs1040337014	G/A	5'UTR	IRES signal
rs577017185	T/A,C	5'UTR	IRES signal
rs750561393	T/A	5'UTR	IRES signal
rs912835892	C/G	5'UTR	IRES signal
rs912835892	C/G	5'UTR	IRES signal
rs1323544073	C/T	5'UTR	IRES signal
rs1264647720	G/T	5'UTR	IRES signal
rs779073537	C/A,T	5'UTR	IRES signal
rs571316178	T/C	5'UTR	IRES signal
rs370124042	G/C,T	5'UTR	IRES signal

**Table 4:** Effect of nsSNPs of TYMP gene on Hydrogenic and hydrphobic interactions.

**dbSNPID**	**SNP**	**Hydrogen bonds**		**Hydrophobic bonds**	
		**Wild type**	**variant**	**Wilde type**	**variant**
rs767829510	T118R	Asp233	Val208 and Asp 233	Ile218, Leu251, Val234, Phe242, Gly119, Val241, Ser117, Asp233 and Thymine	Leu251, Ileu214, Val 241, Asp 209, Phe242 and thymine
rs121913041	R202K	Leu198, Thr205, Thr207, Val208 and thymine	Thr205 and Leu198	Ala 200, Val 204, Ile 214, Thyr199, Leu75, Leu213 and Ser217	Ala 200, Val204, Thr207, Val208, Leu75, Thyr199 and Ser217
rs121913041	R202T	Leu198, Thr205, Thr207, Val208 and thymine	Thr205 and Leu198	Ala 200, Val 204, Ile 214, Thyr199 Leu75, Leu213 and Ser217	Val204, Ala200, Tyr199 Leu75, Thr207, Leu148 Val208
rs1064792867	V208G	Arg202 and Ser210	Arg202 and Ser210	Ala206, Asp203, Leu148 and Ile214	Ile214, Ala206
rs758373793	V241D	-	Thr118, Ile214 and Pro243	--------------------	Thr118, Pro243 and Thymine
rs751803871	S144R	Val185	Thr154 and Val185	Met142, Gln187, Lys221, Thr154 and Ile184	Gln187, Lys221, Met142, Ile184, His116 and Ser126
rs121913037	G145R	Thymine, Tyr199 and Leu155	Thymine, Tyr199 and Leu155	Leu148, Gly153, His116, Thr118 and Gly119	Leu148, Gly153, His116 Thr118 and Gly119
rs188802138	R146H	Tyr199, Glu159 and Asp156	Tyr199 and Asp156	Gly153 and Val166	Gly153 and Leu155
rs188802138	R146S	Tyr199, Glu159 and Asp156	Tyr199	Val166 and Gly153	Gly153 and Asp156
rs121913038	G153S	Asp156 and Lys157	Tyr199, Asp156 and Lys157	Gly147, Arg146, Leu155 and Gly145	Gly147, Gly146, Gly145 and Leu155
rs948237404	V121G	Leu277	Leu277	Asp123, Val281, Ala398 and Pro276	Asp123 and Pro276
rs863224250	G120S	Asp123	Asp123	Thr151, Lys235 and Gly122	Thr151, Lys235 and Gly122
rs863224250	G120R	Asp123	Asp123, Lys275 and Met237	Thr151, Lys235 and Gly122	Thr151, Lys235 and Gly122
rs997046759	G122S	Lys124	Lys124 and Cys280	Gly120, Leu277, Glu286 and Cys280	Gly120, Leu277, Pro276 Glu286 and Val281
rs1388735279	G122D	Lys124	Lys124	Gly120, Leu277, Glu286 and Cys280	Gly120, Cys280, Pro276, Gly278 Val281, Arg279, Leu277 and Glu286
rs1452194925	D123G	Gly120, Lys157, Val125 Lys235 and Ser117	Gly120 and Lys157	Leu277, Val121 and Glu286	Val121 and Val125
rs898036117	R408S	Glu413 and Asp156	------	His402, Val204, Asp203, Leu415 and Arg410	Asp203, Glu413, His402, Val204 and Arg410
rs863224254	G407R	His402	His150 and Ala406	Gly405 and Leu415	Leu415, Gly405, His402, Gly149, Gly152, Thr151 and His283
